# Carotenoid-to-bacteriochlorophyll energy transfer through vibronic coupling in LH2 from *Phaeosprillum molischianum*

**DOI:** 10.1007/s11120-017-0398-3

**Published:** 2017-05-18

**Authors:** Erling Thyrhaug, Craig N. Lincoln, Federico Branchi, Giulio Cerullo, Václav Perlík, František Šanda, Heiko Lokstein, Jürgen Hauer

**Affiliations:** 1 Photonics Institute, TU Wien, Gußhausstraße 27, 1040 Vienna, Austria; 2 Dipartimento di Fisica, IFN-CNR, Politecnico di Milano, Piazza L. da Vinci, 32, 20133 Milan, Italy; 3 Faculty of Mathematics and Physics, Institute of Physics, Charles University, Ke Karlovu 5, 12116 Prague, Czech Republic; 4 Department of Chemical Physics and Optics, Charles University, Ke Karlovu 3, 12116 Praha 2, Czech Republic

**Keywords:** LH2, Ultrafast spectroscopy, Excitation energy transfer, Excitons, Photosynthesis

## Abstract

The peripheral light-harvesting antenna complex (LH2) of purple photosynthetic bacteria is an ideal testing ground for models of structure–function relationships due to its well-determined molecular structure and ultrafast energy deactivation. It has been the target for numerous studies in both theory and ultrafast spectroscopy; nevertheless, certain aspects of the convoluted relaxation network of LH2 lack a satisfactory explanation by conventional theories. For example, the initial carotenoid-to-bacteriochlorophyll energy transfer step necessary on visible light excitation was long considered to follow the Förster mechanism, even though transfer times as short as 40 femtoseconds (fs) have been observed. Such transfer times are hard to accommodate by Förster theory, as the moderate coupling strengths found in LH2 suggest much slower transfer within this framework. In this study, we investigate LH2 from *Phaeospirillum* (*Ph*.) *molischianum* in two types of transient absorption experiments—with narrowband pump and white-light probe resulting in 100 fs time resolution, and with degenerate broadband 10 fs pump and probe pulses. With regard to the split Q_x_ band in this system, we show that vibronically mediated transfer explains both the ultrafast carotenoid-to-B850 transfer, and the almost complete lack of transfer to B800. These results are beyond Förster theory, which predicts an almost equal partition between the two channels.

**Electronic supplementary material:**

The online version of this article (doi:10.1007/s11120-017-0398-3) contains supplementary material, which is available to authorized users.

## Introduction

**Fig. 1:**
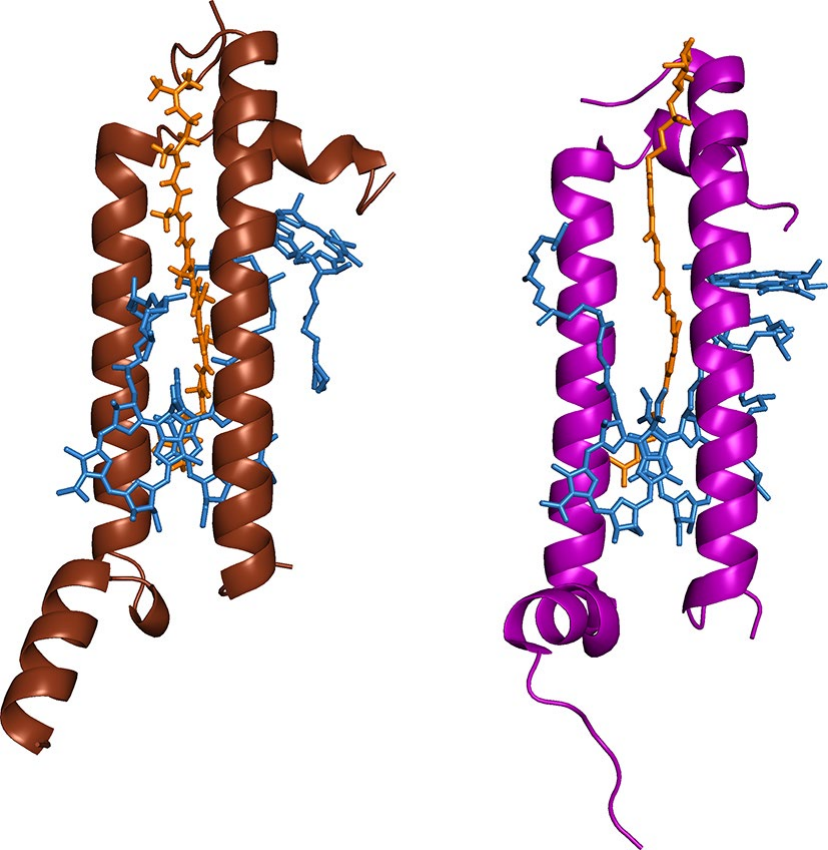
The LH2 α/β heterodimers in the crystal structures of *Ph. molischianum* (*left*) and *Rps. acidophila* (*right*). Bacteriochlorophylls are highlighted in *blue* and carotenoids in *orange*. Structures from the protein data bank (PDB)

In the initial steps of photosynthesis, a pigment is excited by absorption of a solar photon. Through a series of excitation energy transfer (EET) steps, this excitation energy is transferred to a photosynthetic reaction center, where conversion into electrochemical energy takes place (Blankenship 2014). Numerous studies using time-resolved spectroscopy have shown that these initial steps of absorption and EET, occurring in adaptable and versatile pigment–protein complexes, proceed on timescales of tens of femtoseconds to hundreds of picoseconds. These pigment–protein complexes and their interactions are heavily reliant on the interplay of structure and function, and ultimately only through the combination of structural biology and ultrafast spectroscopy one can understand the complex energy transfer network and dynamics in photosynthetic systems. An early example of successfully established structure–function relationship is the peripheral light-harvesting antenna complex LH2 found in purple bacteria. With its crystal structure determined as early as 1995, LH2 from the purple bacterium *Rhodopseudomonas* (*Rps*.) *acidophila* is one of the most intensely studied light-harvesting units (Mcdermott et al. 1995). It consists of a symmetric array of nine α/β apoprotein heterodimers. Each of these units binds three bacteriochlorophyll *a* (BChl) molecules and one carotenoid. In the fully assembled oligomer, 18 of the BChls are incorporated into a strongly coupled ring-like structure, the B850 ring, while the remaining nine BChls form the weakly coupled B800 ring. Even though the overall ring-like structure of LH2 complexes shows a high degree of interspecies similarity, significant deviations are observed upon closer inspection. While in *Rps. acidophila* and *Rhodobacter sphaeroides* ninefold symmetry is observed, LH2 from *Phaeospirillum* (*Ph*.) *molischianum* shows eightfold symmetry, with BChl rings of 16 and eight pigments, respectively, for B850 and B800. Nevertheless, the absorption spectra in the NIR region, where these BChl ring structures show their peak absorption, are almost identical for all these species (Herek et al. 2004). A further distinguishing structural feature between these two complexes, as illustrated in Fig. 1, is the orientation of the B800 ring relative to the B850 ring. While in *Rps. acidophila* the B800 BChl is nearly orthogonal to the B850 BChls, the B800 BChl in *Ph. molischianum* is rotated in plane by 90° and tilted by 38°.

Besides these structural differences, a large variety of carotenoids are bound to LH2 complexes extracted from different organisms (Polivka and Sundstrom 2004). Carotenoids can be described as chain-like polyenes with their photophysical properties to a large extent determined by the number of conjugated double bonds, *N*, ranging from 9 to 13 in naturally occurring bacterial complexes. Most of the bacterial light-harvesting relies on BChls and their excitonic bands, and while carotenoids can also act as an accessory pigment, their primary task is photoprotection, i.e., the quenching of long-lived and potentially harmful BChl triplet states (Blankenship 2014; Niedzwiedzki et al. 2011). Nonetheless, when acting as light-harvesters in the photosynthetic apparatus of purple bacteria, carotenoids have appreciable absorption in the blue–green spectral region of the solar spectrum. Given the steep distance dependence of known EET mechanisms (Valkunas et al. 2013), it is clear that carotenoids need to be in close proximity of BChls for both EET and photoprotection. The structures compared in Fig. 1 illustrate that both rhodopin glucoside (the carotenoid found in *Rps. acidophila*) and lycopene (*Ph. molischianum*) are in van der Waals contact (<5 Å) with the respective B850 and B800 BChls.

EET from carotenoids to BChls may occur from at least two excited states: the lowest lying singlet state S_1_, which is optically dark for transitions from the ground state (S_0_) and the first optically allowed singlet excited state (S_2_). These two states differ not only in the character of their optical transitions from the ground state, but also in their lifetimes of tens to hundreds of femtoseconds for S_2_ and a few to tens of picoseconds for S_1_ (Polivka and Sundstrom 2004). The ultrashort lifetime of S_2_ is explained by relaxation to S_1_, with possible involvement of other intermediate states (Polivka and Sundstrom 2009). Upon excitation of the S_2_ state of a LH2-bound carotenoid, the lowest-energy band formed from BChl Q_y_ transitions in the B850 ring (Q_y_B850) is populated within several 100 fs, which makes S_2_ a likely donor state despite its ultrashort lifetime (Shreve et al. 1991; Macpherson et al. 2001). For *Ph. molischianum*, Rondonuwu et al. (2004) found a 48% EET efficiency from S_2_ to BChl, while the transfer from S_1_ appeared to be negligible. The latter is explained by the S_1_-energy of lycopene (*N* = 11) being 12.500 ± 150 cm^−1^, which hinders transfer to the almost isoenergetic Q_y_B800 band, while direct transfer to Q_y_B850 is unfavorable due to a mismatch of absorption and fluorescence spectra (Billsten et al. 2002a). The same reasoning applies to rhodopin glucoside, which also has 11 double bonds (Polivka et al. 2002).

To describe the EET process from S_2_ to the bands formed by BChl Q_x_ transitions, given the moderate coupling of approximately 110 cm^−1^ (Tretiak et al. 2000; Damjanovic et al. 1999), theoretical approaches grounded in perturbative treatment of coupling (Förster theory) have been employed. However, even using sophisticated ab initio estimates of couplings (e.g., the transition density cube method), Förster theory tends to overestimate S_2_-to-Qx transfer times (Tretiak et al. 2000), which can be as fast as 40 fs for LH2 from *Marichromatium purpuratum* (Polli et al. 2006) or even 26 fs in the main light-harvesting pigment–protein complex of green plants (LHCII) (Knox 1999). As such, these extremely fast transfer times seem incompatible with the traditional framework of Förster theory.

Recently, Perlik et al. (2015) described how vibronic coupling successfully explains both dynamical and static spectral properties of a donor–acceptor system closely mimicking the properties of S_2_ (carotenoid) and Q_x_ (BChl). In this vibronic model, an underdamped vibration located at the carotenoid (donor) was treated on the same footing as electronic degrees of freedom in the system Hamiltonian. This allowed for a discrete vibrational structure of the electronic ground and excited state manifold. Resonance between the donor–acceptor energy gap and transitions to vibronically shifted levels on electronic ground and excited states allowed for dissipation of excess energy during carotenoid-to-BChl transport to the ground-state vibration, which explained the observed ultrafast transfer rates.

**Fig. 2:**
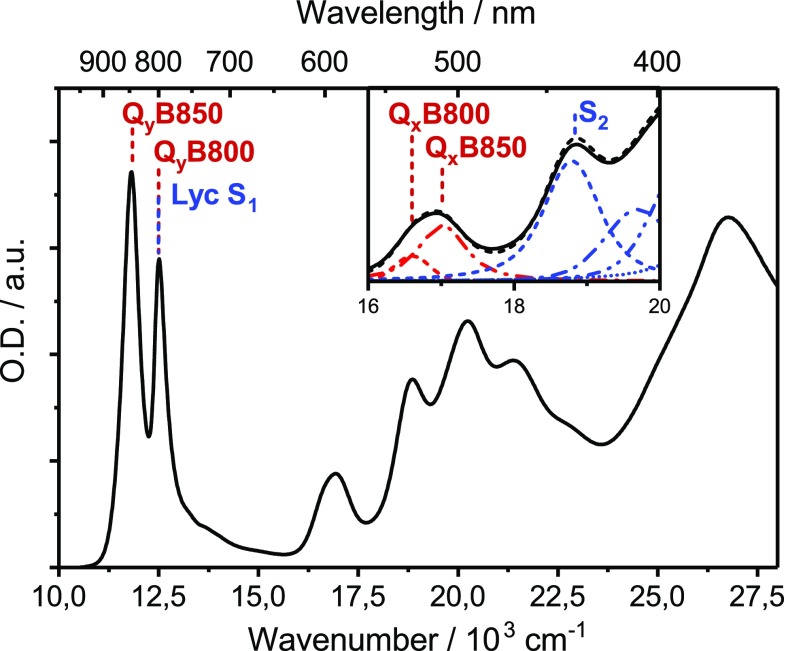
Normalized absorption spectra of LH2 from *Ph. molischianum* in buffer solution at room temperature. The Q_y_ bands associated with the B800 and B850 rings are labeled in *red*, while the approximate position of the lycopene S_1_ transition in solution is indicated in *blue. Inset*: Magnified view of the BChlQ_x_ transition region from 16.000 to 20.000 cm^−1^. The absorption profile can be fit to a “split” Q_x_ band (*red*) and a series of vibronic bands related to the lycopene S_2_ absorption (*blue*)

The “split” Q_x_ band of *Ph. molischianum* represents an ideal test case for the vibronic coupling model: as the coupling constants between S_2_ and both Q_x_ bands (Q_x_B800 and Q_x_B850 in Fig. 2) are known (Tretiak et al. 2000), the predictions of this model can be tested for two independent EET channels. To this end, we perform two transient absorption experiments: in the first set of experiments, we excite S_2_ and use visible- and NIR-supercontinuum probe to study the entire EET network with sub-100 fs time resolution. To accurately characterize the S_2_-to-Qx EET dynamics, which have been observed to proceed on timescales well below 100 fs, we additionally perform sub-10 fs transient absorption experiments with visible pulses. By combination of both experiments, along with global (target) analysis, we arrive at a comprehensive picture of energy flow dynamics in *Ph. molischianum*. We further show that the vibronic coupling model, applied to the LH2 complex, is able to explain both the rate and the directionality of the EET process.

## Results and discussion

The absorption spectrum of LH2 from *Ph. molischianum,* shown in Fig. 2, is typical for purple bacterial LH2 complexes. Two sharp excitonic transitions, Q_y_B850 and Q_y_B800, formed from Q_y_ transitions of the two BChl rings are observed in the near-infrared. The associated out-of-plane BChlQ_x_ transitions appear as broad and relatively weak features around 16.500 cm^−1^ (~600 nm), and show properties similar to monomeric BChl (Blankenship 2014). In most LH2 complexes, e.g., from *Rps. acidophila* or *Rb. sphaeroides*, the Q_x_ transitions are essentially degenerate, resulting in a close-to-symmetric absorption band (Rondonuwu et al. 2004; Billsten et al. 2002a; Cong et al. 2008; Christiana et al. 2009). In contrast, the Q_x_ band of *Ph. molischianum* is clearly asymmetric, and is best described by two overlapping transitions with slightly different central frequencies (17.030 and 16.600 cm^−1^), as demonstrated in the inset of Fig. 2. The higher energy of these transitions contains approximately twice the oscillator strength of the lower transition, suggesting that it is the Q_x_ band associated with the B850 ring. This energetic splitting suggests that the Q_x_ transitions of B800 and B850 BChls experience different coupling to the surrounding protein in LH2 of *Ph. molischianum*, while the two environments appear to be equivalently polar for *e.g., Rps. acidophila*. Indeed, one finds that a more polar aspartate residue is the Mg ligand in *Ph. molischianum* but a formyl-methionine in *Rps. acidophila* (Koepke et al. 1996).

At excitation energies higher than approximately 18.000 cm^−1^ in the visible range, optical transitions are assigned predominantly to the progression of carotenoid S_0_–S_2_ vibronic states. As coupling between carotenoids and BChls in LH complexes is relatively weak, excited states are largely localized on one type of pigment. The ~1000 cm^−1^ red-shift of the carotenoid absorption observed upon incorporation into LH2 is thus not due to electronic coupling, but rather interaction with the protein pocket (Herek et al. 2004). As such, excitation below approximately 18.000 cm^−1^ (555 nm) will result in direct excitation of BChl states, while in the 18–22.000 cm^−1^ range excitation will predominantly result in population of excited states of lycopene.

## Excitation energy transfer dynamics

**Fig. 3:**
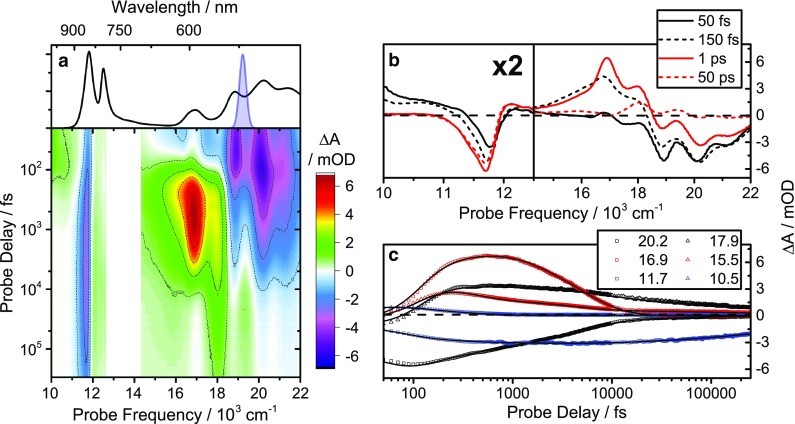
**a** Linear absorption spectrum and 2D time/frequency transient absorption map following excitation of the lycopene S_0_–S_2_ transition. The excitation pulse spectrum is indicated by the *blue* shaded area. **b** Transient absorption spectra at selected delay times. **c** Kinetics at selected probe frequencies (*symbols*) overlaid with fits from a global exponential decay model (*solid lines*). Units are in 10^3^ cm^−1^

In order to investigate the relaxation processes in LH2 from *Ph. molischianum* after carotenoid excitation, we perform a series of transient absorption (TA) experiments. In Fig. 3a the TA spectra using narrowband excitation and supercontinuum probe (see “Experimental” section for details) are presented as a two-dimensional probe frequency versus time map. The overall relaxation dynamics are multi-exponential and involve decay times spanning from tens of femtoseconds to hundreds of picoseconds. The relatively weak BChl-lycopene coupling, however, enables us, to a good approximation, to analyze the complex data in terms of transitions associated with lycopene- and BChl-related states. In particular, it allows us to use the response of isolated lycopene as a reference in the interpretation of relaxation dynamics, greatly facilitating the task of assigning spectral signatures to electronic states.

**Fig. 4:**
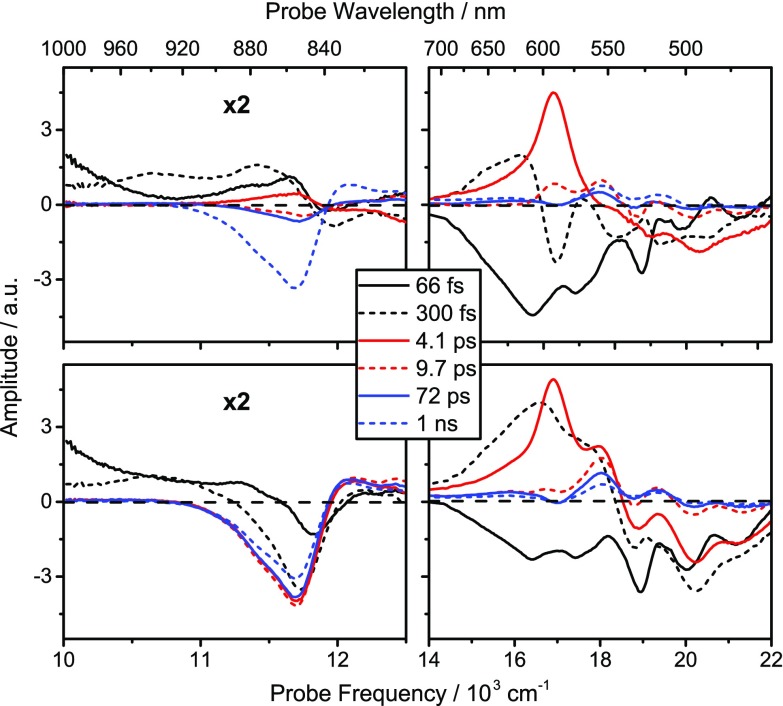
Decay-associated spectra of LH2 from *Ph. molischianum* in the near-infrared (*left*) and visible (*right*) spectral region. Note the rescaling of the near-infrared amplitudes. *Top* DAS (parallel decay model), *bottom* EADS (sequential decay model). The amplitude of the 66 fs component in all panels is divided by 2 in order to facilitate comparison of spectral shapes between components

To aid this assignment, we perform global analysis on the data, using a total of six exponential components to describe the dataset (Fig. 4). The principles of global analysis have been explored in great detail elsewhere (Lincoln et al. 2016; van Stokkum et al. 2004). We show the decay-associated spectra (DAS, parallel decay model) and the evolution-associated differential spectra (EADS, sequential model) in Fig. 4. The quality of the fit can be assessed in Fig. 3c, and the spectra retrieved from the sequential model (evolution-associated difference spectra, EADS) are in good agreement with the previously reported spectra (Rondonuwu et al. 2004; Billsten et al. 2002a, b).

In short, in the 14–22.000 cm^−1^ region essentially all spectral features are attributable to lycopene localized states. The first bright state, the S_2_ state of lycopene, has a lifetime of 66 fs and exhibits strong ground-state bleach (GSB) and stimulated emission (SE) features. This is followed by the vibrationally hot S_1_ state (hot-S_1_), which cools on a 300 fs timescale to the equilibrated S_1_ state. The lifetime of this state, 4.1 ps, is in good agreement with the literature values for lycopene in solution (Kosumi et al. 2009; Billsten et al. 2002b). Both of these states show strong excited state absorption (ESA) contributions between 14 and 19.000 cm^−1^ in addition to the S_2_ GSB. The following three retrieved spectra, with time constants 9.7, 72 ps and 1 ns, all exhibit an ESA feature at 18.000 cm^−1^ and only a weak S_2_ GSB contribution. This feature, which can also be seen in the hot-S_1_ and S_1_ spectra, has been assigned to a carotenoid triplet, a triplet precursor, the so-called S* state, recently to a field induced shift of the S_2_ GSB (Herek et al. 2004; Niedzwiedzki et al. 2016) and combinations thereof. A full analysis of the feature is beyond the scope of this paper, and we will from here on refer to it as S* (Gradinaru et al. 2001; Christensson et al. 2009; Balevičius et al. 2015, 2016). Additional features attributed to the carotenoid are two ESA bands in the 10–12.000 cm^−1^ region. One of these, having a 66 fs lifetime, is assigned to S_2_-to-S_m_ transitions (Christensson et al. 2010), while the second with lifetime of 300 fs is tentatively assigned to hot S_1_-to-S_n_ transitions (Andersson and Gillbro 1995).

Beyond these minor ESA features, the 11–13.000 cm^−1^ NIR region of the spectrum is dominated by BChl features, in good agreement with earlier reports (Ihalainen et al. 2001). In both the early-time raw data (Fig. 3c) and in the first 66 fs components in the global decay models (Fig. 4), there is a pronounced GSB corresponding to the Q_y_B850 absorption. We attribute this to excitation of high-lying states associated with the B850 ring (Q_x_ or B), and their strong coupling to Q_y_B850. The following time component (300 fs) spectra are significantly red-shifted, with a weak ESA feature towards the blue edge. This is due to a population appearing in the Q_y_B850 band (Rondonuwu et al. 2004), resulting in SE and ESA features related to the band in addition to the already present GSB. Hence the rapid buildup of the excited state population in the Q_y_B850 band proceeds on a timescale of 100–300 fs. On longer timescales, illustrated by the spectra at 1 ps (Fig. 3c) and the 4.1 ps global decay component (Fig. 4), we can observe only a minor increase in this feature. This implies that Q_y_B850 excited state population is predominantly formed by transfer from S_2_, rather than the slower channels involving transfer via S_1_ states. The concomitant Q_x_B850 GSB signal is almost impossible to see without de-convolving the spectra into their constituent components due to the much stronger opposing ESA from the carotenoid. One can see it beginning to appear for delays longer than 50 ps though it is still not fully resolved.

Importantly, we find no indication of significant Q_y_B800 population during the relaxation process. This could partly be due to its transient nature, as it is known to relax to Q_y_B850 on *a* ~1 ps timescale (Billsten et al. 2002a; Theiss et al. 2008), and to the spectral overlap with Q_y_B850 ESA. Notwithstanding, it remains less than expected from other LH2, e.g., *Allochromatium vinosum* (Niedzwiedzki et al. 2012) and *Rb. sphaeroides* (Cong et al. 2008).

The Q_y_B850 formation kinetics substantiates this observation. In LH2 from *Allochromatium vinosum* and *Rb. sphaeroides*, the formation dynamics of the Q_y_B850 band has multiple components, corresponding to internal Q_x_B850-to-Q_y_B850 relaxation, inter*-*ring Q_y_B800-Q_y_B850 EET, and transfer from the carotenoid S_1_ state (likely via the Q_y_B800 band) (Cong et al. 2008). Although the latter two processes are significant in the dynamics of these LH2 complexes, they are relatively slow, with time constants in the range of approximately 800–1000 fs and 4–5 ps, respectively (Macpherson et al. 2001; Billsten et al. 2002a; Polivka et al. 2007) depending on the organism. In stark contrast, the Q_y_B850 kinetics displayed in Fig. 3 are simple and strongly dominated by the 66 fs buildup and the slow mono-exponential ground-state recovery on a timescale long compared to the time window probed here. The implication of this observation is that neither the S_2_-to-B800 nor the S_1_-to-B800 relaxation channels contribute significantly to light-harvesting of the complex upon green-light excitation. Overall, these observations strongly suggest that the initial S_2_ population transfers, with essentially equal probability, either to states related to the B850 ring or to the S_1_ state. As S_1_-to-BChl transfer is inefficient, it appears that on “green-light” excitation, neither lower-energy carotenoid states nor the B800 ring contributes to functional light-harvesting (Damjanovic et al. 1999).

## Ultrafast dynamics in the carotenoid/Q_x_ spectral region

The narrowband pump–supercontinuum probe studies described above demonstrate that, within the limited time resolution provided by the experiment, the effective carotenoid S_2_-to-Q_x_B850 transfer proceeds on a timescale of 100 fs or faster. Moreover, the Q_y_B800–Q_y_B850 and S_1_-Q_y_B850 transfer channels present in LH2 from a number of other organisms (Mirkovic et al. 2017) are also clearly shown to be largely insignificant in visible range light-harvesting in *Ph. molischianum*. However, since the timescale of the rapid rise of Q_y_B850 is considerably shorter than the instrument response function, it was not possible to reliably assess the underlying mechanism behind the suppression of the S_2_-Q_y_B800 channel.

**Fig. 5:**
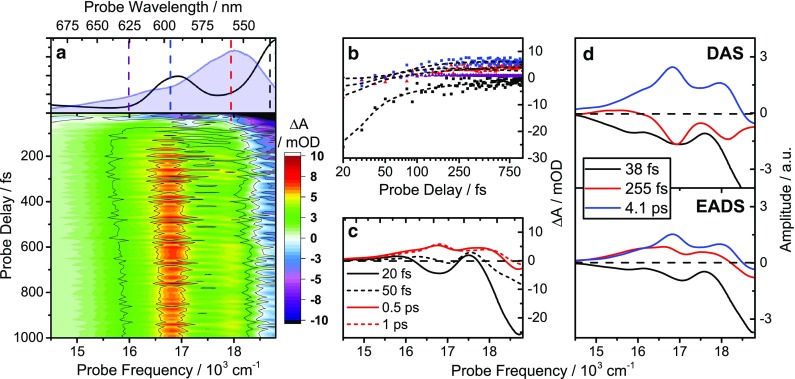
**a** Linear absorption spectrum (*black line*) and laser spectrum (shaded *blue* area), and 2D time/frequency transient absorption map. **b** Transient absorption kinetics (*symbols*) overlaid with the traces from a global fit to the data (*dashed lines*) at the probe frequencies marked with *vertical dashed lines* in (**a**). **c** Transient absorption spectra at selected probe delays. **d** Decay-associated spectra (see text for details). The amplitude of the 38 fs component has been halved to facilitate the comparison of spectral shapes

In order to characterize the initial carotenoid relaxation, we perform broadband femtosecond TA using ~10 fs pulses centered around 17.500 cm^−1^. Figure 5 shows the employed laser spectrum along with the corresponding LH2 absorption spectrum. The population relaxation dynamics are in good qualitative agreement with the results from the narrowband experiment, with preserved spectral lineshapes for S_2_ GSB and SE, hot-S_1_ ESA, and the cooled S_1_ ESA with a pronounced S* feature (Fig. 5). The retrieved lifetime for S_2_ is considerably shorter than that from narrowband excitation however, and we observe also the GSB and SE features related to the Q_x_BChl bands at early times due to their direct excitation by the broadband pulse.

The 38 fs time constant for carotenoid S_2_ decay in LH2, as opposed to 110 fs in lycopene in hexane (Billsten et al. 2002b), implies that the total time constant for S_2_-to-BChl transfer is approximately 60 fs, in agreement with the observation of Q_y_B850 population within the instrument response in the narrowband experiment. Such fast carotenoid-to-BChl transfer times have been reported also for other light-harvesting antenna systems (Polli et al. 2006; Perlik et al. 2015); however, they are too fast to be reconcilable with dipole–dipole coupling assuming a Förster mechanism (Krueger et al. 1998). It was suggested that the involvement of vibrations and vibronic mixing is of importance for EET processes in LH2 (Perlik et al. 2015). Indeed, our experimental observations suggest that vibrational motion is significantly involved in the energy transfer dynamics, as explained in the following section.

## Coherent dynamics in the carotenoid—Q_X_ spectral region

**Fig. 6:**
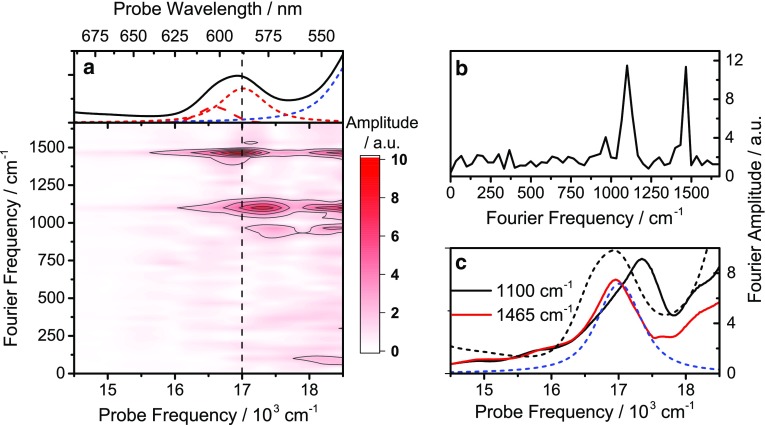
**a** Absorption spectrum (*top*) and Fourier transform amplitudes of the residuals after subtraction of exponential population dynamics as a function of probe and Fourier transform frequency. **b** Integration of the data in **a** over all probe frequencies yields the impulse stimulated Raman spectrum. **c** Probe frequency dependence of the amplitudes of the two dominating modes (*solid lines*). The total absorption spectrum and the Q_x_B850 absorption (*black* and *blue dashed lines*) are superimposed for comparison

Clear and persistent oscillations—vibrational *quantum beats* (QBs)—in the kinetics can be observed at several probe energies after broadband excitation. Analyzing these signals, assigning their physical origin, and determining their (possible) relevance to biological function are some of the major challenges in current research into light-harvesting mechanisms (Fuller et al. 2014). We proceed by isolating the oscillatory components by subtraction of the globally fitted population dynamics from the raw data leaving only purely oscillatory residuals, similar to the work of Lincoln et al. (2016). A Fourier transform of the delay time axis of these residuals yields the 2D map shown in Fig. 6a, where the QB amplitude is plotted as a function of QB frequency and probe frequency. From this map, we can learn not only which QB frequencies are present (and their relative intensities), but also at which probe energies they contribute to the signals. From the integrated amplitude spectrum (IAS) shown in Fig. 6b, generated by integrating the map in Fig. 6a over all probe energies, we identify two particularly prominent modes. These appear with frequencies of ~1100 and ~1465 cm^−1^, and can be assigned to well-known ground-state modes of carotenoids (Merlin 1985) though with an approximately −50 cm^−1^ offset. We note that the IAS is related to the resonance Raman spectrum in the case where QBs originate primarily from ground-state contributions, although the relationship is not entirely trivial (Liebel et al. 2015). Plotting the amplitude of these vibrational modes individually as a function of probe energy yields the spectrum in Fig. 6c. For both modes, we observe two amplitude maxima: one approaching the S_2_ absorption maximum and one red-shifted by the vibrational frequency relative to a zero-phonon line estimated to be near 18.400 cm^−1^ (see SI). This is exactly the amplitude pattern expected from consideration of ground-state coherent pathways (i.e., impulsive stimulated Raman scattering) in this system (Lincoln et al. 2016; Jumper et al. 2016). The crucial observation for interpretation of the physical significance of these modes, beyond them being simple ground-state vibrations, is the overlap between the lower-energy amplitude maximum of the 1465 cm^−1^ mode and the Q_x_B850 band. As a result, the strongly allowed carotenoid S_2_–S_0_ (0–1) transition is in essentially perfect resonance with the Q_x_B850 transition. This is a requirement for the vibronically mediated transfer mechanism described in detail elsewhere (Perlik et al. 2015), which will be summarized and applied to LH2 from *Ph. molischianum* in the following section.

## The mechanism of excitation energy transfer

Both low- and high-time resolution TA experiments consistently indicate ultrafast relaxation of the carotenoid S_2_ state in conjunction with strongly preferential sub-100 fs population of states associated with the B850 ring. These observations stand in—both quantitative and qualitative—contrast to the calculated relaxation dynamics in this LH2 complex (Tretiak et al. 2000; Damjanovic et al. 1999), where transfer to the B800 and B850 rings was suggested to be approximately equal and to proceed on timescales of 200–300 fs. Notably, a common feature of the models used in these earlier theoretical studies is that they rely on essentially Förster-type resonant EET. The TA data, on the other hand, suggest that vibrational states may have non-trivial involvement in the EET process. As vibronic models typically predict significant speed up of EET around excitonic-vibrational resonance as compared to Förster, explicitly including the vibrations in the model thus appears necessary.

**Fig. 7:**
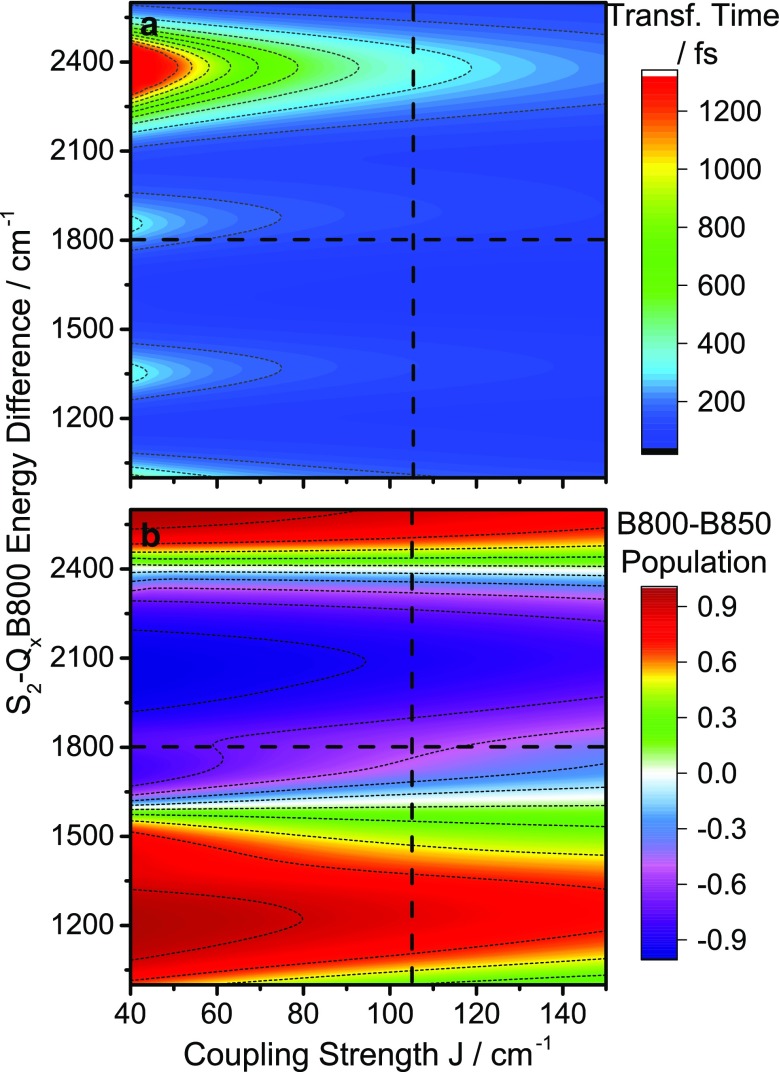
**a** S_2_ to BChlQ_x_ transfer time as a function of level splitting and coupling strength (See text for details) **b** The normalized population difference between the B800 and B850 band at the transfer time *t* (where the population of S_2_ is reduced to 1/*e* of its initial value), as a function of level splitting and coupling strength. *Dashed lines* correspond to the observed carotenoid S_2_-B800 level splitting and the coupling strength used in our calculations

In a recent study, Perlik et al. (2015) showed that ground-state vibrations in a synthetic mimic of the LH2 complex heavily influence the observed EET rates under the appropriate resonance conditions. We here apply the vibronic approach (Perlik and Sanda 2016) to LH2 itself by extending the exciton part of the model in Perlik et al. (2015) to contain the two Q_x_ states associated with the BChl rings along with the S_2_ state of the carotenoid (see supporting information and Perlik and Sanda 2016 for details). Further, in order to investigate the possible contributions of vibrational states, we explicitly include both of the strongly active ground-state vibrational modes at ~1500 and ~1100 cm^−1^ observed in TA experiments. The energy difference between Q_x_B850 and Q_x_B800 is fixed to the experimental value, and we calculate the S_2_-to-Q_x_ transfer time (defined as the time needed for the S_2_ population to reach 1/e its initial size) as a function of lycopene-BChl coupling *J* and the S_2_-Q_x_B800 energy detuning *dE*. The results of the calculation are depicted in Fig. 7a.

The dependence on coupling strength is simple, showing a monotonic decrease of transfer time with increasing *J* as expected. The energy detuning dependence, on the other hand, shows much more complex behavior, with alternating regions of fast and slow transfer. This complex dependence on *dE* can be understood by identifying the most prominent relaxations pathways: In both experiment and model the majority of initial S_2_ population is generated close to the zero-phonon line, and excitation energy is subsequently transferred from this vibrationally cold S_2_ state downwards to the energetically closest BChl eigenstate. Depending on *dE*, the eigenstate nearest to the vibrationally relaxed S_2_ can be localized on either B800 or B850 (see SI Fig. 1). Minima along the *dE* axis of Fig. 7a (i.e., regions of fast transfer) appear as the initially excited S_2_ state of lycopene comes into resonance with a state containing electronically excited BChl Q_x_ *and* excited carotenoid ground-state vibrations (Perlik et al. 2015).

Applying the experimentally observed energy levels splitting and the lycopene-BChl coupling strengths calculated by Tretiak et al. (2000) as parameters in the model lets us identify the expected transfer times for LH2, denoted by dashed lines in Fig. 7a. With these parameters, the S_2_ state is close to resonant with Q_x_B850 and a 1500 cm^−1^ vibration, while all Q_x_B800 eigenstates are far out of resonance. As a result, transfer is strongly directional towards the B850 ring, as shown in Fig. 7b. The calculations result in a transfer time of approximately 100 fs and a branching ratio of approximately 3.5 in favor of Q_x_B850 transfer. This is close to the experimental observations, thus showing that the theoretical approach provides a plausible mechanism to explain both the extremely fast S_2_-to-BChl transfer and the strong preference for transfer towards the B850 ring.

## Conclusions

We employed visible pump, visible/NIR white-light probe spectroscopy to monitor the entire carotenoid-to-BChl energy transfer in LH2 from *Ph. molischianum* and found that the B800 ring is almost entirely avoided in the energy deactivation network of the complex. To further investigate this intriguing finding, we used sub-10 fs pulses for both pumping and probing and observed extremely fast transfer from S_2_ to QxB850. The analysis of pronounced vibrational quantum beats in the high-resolution experiment along with theoretical modeling allowed us to relate this unusual behavior to the influence of ground-state vibrational modes, located solely on the carotenoid. After the role of vibronic coupling on S_2_-to-Q_x_-transfer has been clarified in this study, future work will focus on high-time-resolution visible excitation-NIR probe experiments. These may further clarify whether or not the ultrafast Q_x_-to-Q_y_-transfer [44 fs in LH1 from *Rhodospirillum rubrum* (Maiuri et al. 2015) is vibronically mediated.

## Experimental

### Sample preparation

*Phaeospirillum* (*Ph*.) *molischianum* was cultured strictly anaerobically at 30 °C under incandescent illumination. Cultures were harvested by centrifugation and resuspended in 50 mM TRICIN buffer (pH 8.0). Cells were broken in a FRENCH press at 950 PSI. Membranes were resuspended in 20 mM TRICIN-buffer (pH 8.0) to an OD of 50 at 846 nm. The detergent β-dodecyl maltoside (β-DDM, GLYCON, Luckenwalde, Germany) was added to 0.5%; the suspension was incubated with shaking at RT for 20 min and centrifuged to remove undissolved membranes. The supernatant was loaded onto a 25–10% sucrose gradient, containing 0.03% β-DDM and ultracentrifuged at 130.000 g for 16 h (overnight) at 4 °C. Two major brownish bands were visible, the upper one being LH2. The LH2 fraction was concentrated in CENTRICON 100 devices at 20 °C and 3.000 g to remove small(er) proteins. Further purification was carried out by another sucrose gradient and/or size exclusion chromatography on a column equilibrated with 20 mM TRICIN-buffer (pH 8.0) and 0.03% β-DDM.

### Spectroscopy

The broadband pump–probe set-up (Polli et al. 2007) is based on an amplified Ti:sapphire laser system (Libra, Coherent), which delivers a 2 kHz pulse train of 4-mJ, 100-fs pulses at 800 nm. A portion of the laser output was used to pump an optical parametric amplifier (OPA): a narrowband pulse (10 nm FWHM, 15 nJ/pulse, centered at 520 nm) is obtained by amplification of a spectrally filtered white-light continuum generated in a 2 mm Sapphire plate. The pump for this process is the second harmonic of a part of the 800 nm beam. The white-light continuum probe pulse was generated by focusing a small fraction of the laser fundamental in a 2-mm sapphire plate. Pump and probe beams were synchronized by a delay line and focused on the sample by a 500-mm focal length lens and a 200-mm focal length spherical mirror, respectively. The pump beam was blocked after the sample by an iris. The total instrument response function of the system was approximately 120 fs FWHM, largely limited by the pump pulse duration.

The ΔT/*T* differential signal was obtained by dispersing the probe pulse on an optical multichannel analyzer and subtracting pump on and pump off spectra. A 2D map of the ΔT/*T* signal versus probe delay was obtained by stacking the signal acquired for different delays τ between pump and probe pulses.

The broadband femtosecond transient absorption data were collected using heterodyned transient grating as described previously (Lincoln et al. 2016) with the exception that the data were collected along a single phase matching direction and phased to pump probe data. The pulse spectrum, as shown in Fig. 6a, was compressed to 10 fs duration, using a combination of a prism pair and a grating compressor in a 4f design.

## Electronic supplementary material

Below is the link to the electronic supplementary material.

Supplementary material 1 (PDF 993 KB)
