# Supplementary material for: Carotenoid-to-bacteriochlorophyll energy transfer through vibronic coupling in LH2 from Phaeosprillum molischianum
Source: Photosynth Res. 2017 May 18;135(1):45–54. doi: 10.1007/s11120-017-0398-3 (PMC5783993; doi:10.1007/s11120-017-0398-3)
Supplement: Supplementary file 1 — Supplementary material 1 (PDF 993 KB) [file 11120_2017_398_MOESM1_ESM.pdf]

Supporting Information for:

Excitation energy Transfer through Vibronic Coupling in LH2 from *Phaeosprillum molischianum*

*Erling Thyrhaug[1], Craig N. Lincoln[1], Federico Branchi[2], Giulio Cerullo[2], Václav Perlík[4], František Šanda[4], Heiko Lokstein[3], Jürgen Hauer[1]**

[1] Photonics Institute, TU Wien, Gußhausstraße 27, 1040 Wien, Austria

[2] IFN-CNR, Dipartimento di Fisica, Politecnico di Milano, Piazza L. da Vinci, 32, 20133 Milano, Italy

[3] Department of Chemical Physics and Optics, Charles University, Ke Karlovu 3, CZ-121 16 Praha 2, Czech Republic

[4] Institute of Physics, Faculty of Mathematics and Physics, Charles University, Ke Karlovu 5, 121 16 Prague, Czech Republic

* juergen.hauer@tuwien.ac.at

TRANSFER RATE CALCULATIONS

As detailed in the main text, we observe in transient absorption experiments ultrafast carotenoid S_2 relaxation in conjunction with strongly preferential sub-100 fs population of states associated with the B850 ring. This stands in contrast, both quantitative and qualitative, to relaxation dynamics calculated within the framework of Förster theory in this LH2 complex (Damjanovic et al. 1999; Tretiak et al. 2000). In these studies transfer to the B800 and B850 rings was calculated to be approximately equal and to proceed on timescales of 200-300 fs.

We here apply an extension of the approach of Perlik *et al.*(Perlik et al. 2015) in order to analyze the energy transfer in *Ph. molischianum*. The coupled carotenoid-bacteriochlorophyll system of (Perlik et al. 2015) is modified to include all constituent parts relevant to the early-time carotenoid-to-BChl transfer in *Ph. molischianum*. As we focus on early relaxation dynamics on sub 100 fs timescale, we can safely neglect other relaxation channels than the S_2-to-Q_x transfer, given that these proceed on much longer timescales.

To model the initial transfer we define the system to include a carotenoid and two BChl states. The excitations on the carotenoid are reduced to a S_2 electronic state, with a transition frequency ϵ_{lyc}, and two underdamped vibrational modes of frequencies ω_1 =1530 cm^{-1} and ω_2 =1150cm^{-1}. The Hamiltonian for the lycopene is

eq 1. $$H_{lyc} = \sum_{i=1}^{2} T_i + V_i(q_i) + \left(\epsilon_{lyc} - \sum_{i=1}^{2} m_i\omega_i d_i(q_i - d_i/2)\right)|S2\rangle\langle S2|,$$

where $T_i = \frac{p_i^2}{2m_i}$ is the kinetic energy and $V_i = \frac{m_i\omega_i q_i^2}{2}$ is the potential energy of a harmonic oscillator, and d_i is the displacement of the ground- and excited- oscillator surfaces. The excitonic manifold of the B800 and B850 BChls is modeled as two levels with Hamiltonian

eq 2. $$H_{bchl} = \epsilon_{B800}|B800\rangle\langle B800| + \epsilon_{B850}|B850\rangle\langle B850|,$$

and is further coupled to the lycopene by resonant interaction

$$\text{eq 3.} \qquad H_{coupling} = J\,[|S2\rangle\langle B850| + |B850\rangle\langle S2| + |S2\rangle\langle B800| + |B800\rangle\langle S2|].$$

The excitonic Hilbert space is thus composed of four electronic levels, ground state, and the three excited states S2, B800, B850 and combined with (Fock) states $|n\rangle;\ n = 0,1,\dots$ of the two vibrations $q_1,\ q_2$. The total Hamiltonian $H_{lyc} + H_{bchl} + H_{coupling}$ has been diagonalized in the combined space to obtain vibronic states.

The environmental fluctuation of transition frequencies and vibrational damping are modeled by linear coupling to a harmonic bath with overdamped spectral density [details of the model given in (Perlik and Sanda 2016)] and parameterized by fitting the lycopene and BChl Qx band absorption spectrum. With this full parameterization of the carotenoid-BChl dimer we can investigate the relaxation dynamics using the quantum master equation of (Perlik and Sanda 2016). We obtain an initial exciton population distribution relevant to the experiment by weighting the transition dipole moments by the excitation pulse frequency profile, shown overlaid on the LH2 absorption spectrum in Figure 5a in the main text.

We proceed by solving the master equation, and calculate transport dynamics and transfer times (defined as the time when the excited lycopene population has decayed by transfer to BChl states to 1/e of its initial population). We then analyze the transfer time dependence on S_2- Q_xB800 energy detuning and on the lycopene-BChl coupling *J*. The results of the calculation are depicted in Figure 7 in the main text.

In the calculation, we fix the energy difference between Q_xB850 and Q_xB800 according to the fit to the absorption spectrum in Fig. 2 of the main text and vary the carotenoid-BChls energy-difference parameter *dE*:

eq 4. $dE = \epsilon_{lyc} - \epsilon_{B850}$.

As the majority of excitation is transferred from vibrationally cold S_2 ($|S2\ 0_{1150}\ 0_{1530}\rangle$) to the energetically closest lower BChl eigenstate, we can understand the *dE* dependence by considering the energy diagram for vibronic –levels. Depending on *dE* the nearest lower eigenstate with respect to the S_2 state can be localized either on Q_xB800 or Q_xB850, as can be seen from Figure 1. Here the black line depicts the S_2 energy (relative to Q_xB800) at decreasing values of *dE* intersecting level lines when in resonance. From this qualitative picture one can understand subsequent minima (i.e. regions of fast transfer) along the *dE* axis on Figure 7 of the main text: these occur when the initially excited S_2 state of lycopene are in resonance with BChl Qx states plus carotenoid's ground state vibrations $\epsilon_{B800,B850} + n\omega_1 + m\omega_2$, where $(n, m = 0,1,2, \ldots)$. We note that from the B850-dimer in the α/β subunit only one BChl has appreciable coupling to S_2, which justifies our trimeric treatment of the carotenoid-to-BChl energy transfer.

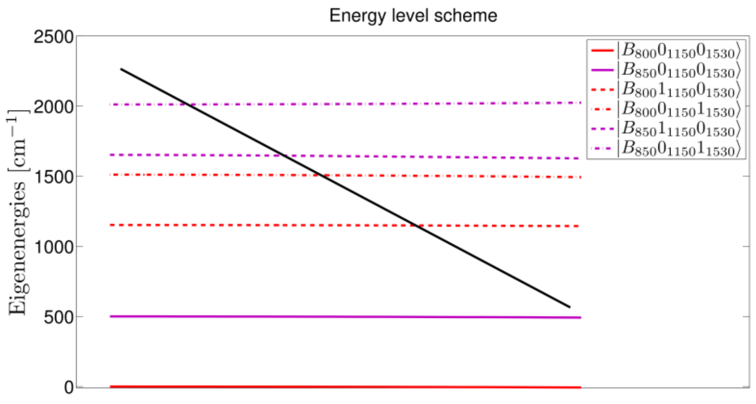

Figure 1: Vibronic level scheme of the single excited state manifold of the model trimer, zero energy is set for the lowest eigenstate $|B_{800}\ 0_{1150}\ 0_{1520}\ \rangle$, carotenoid-Bchl energy difference *dE* is varied Transfer is favored to the nearest lower state from the $|S_2\ 0_{1150}\ 0_{1520}\ \rangle$.

GLOBAL FIT DEFINITIONS

Two standard global analysis approaches are taken in the text, which we denote as: The *decay associated spectra* (DAS) and the *evolution associated difference spectra* (EADS). The fundamentals of global analysis in time-resolved spectroscopy have been described in great detail elsewhere (van Stokkum et al. 2004), however we briefly summarize a few key aspects here. The DAS procedure is the simplest, “naïve” global fitting procedure. The kinetics at every detection frequency are simultaneously fit to a sum-of-exponentials model, with a global constraint on the component decay times. The result is a description of the time-evolution of the transient spectra in terms of a number of components (six in total in the current dataset), each of which consists of a detection frequency dependent amplitude and a characteristic exponential decay time.

Simply plotting the amplitude (which may be positive or negative in TA experiments) associated with a given decay time as a function of probe frequency yields the *Decay Associated Spectrum* (DAS). The EADS procedure is to a large extent similar (and the same characteristic time-constants are extracted); however, a specific relaxation model is imposed on the data as an

additional constraint: it is assumed that each component represents the decay of a specific electronic state, which "feeds into" the next state in a ladder-like fashion. Because of this, it is often referred to as the "sequential" model, and can be thought of as the simplest global target model. The resulting spectra can be interpreted as the spectra of the individual states in a sequential relaxation model, which, while not necessarily directly representative of the actual physics of the system, will often give useful information about its time-evolution.

GLOBAL TARGET MODELLING OF THE LH2 DYNAMICS

We fit the data to several target models to the data, with the most realistic stable model being shown in Figure 3. This model is likely somewhat simpler than the actual physical system, but presents an effective model of the most strongly contributing processes in this LH2 complex. The retrieved S_2-to-xxx, where xxx = hot-S_1, B800 and B850 relaxation rates (Table 1) give branching ratios of 35%, 13%, 52%, respectively. If one sums the B800 and B850 contributions the resultant 65% yield of S_2-to-B850 is high compared to the 48% measured by Koyama and coworkers (Rondonuwu et al. 2004). One contributing factor could be the absence of S_2-to-^{3}Car and ground state recovery relaxation channels. Inclusion of these relaxation channels results in failure of the fit however, and their absence results in an over-estimate of the S_1 lifetime and under-estimation of the Q_yB850 lifetime. S_1-to-BChl transfer channels were also tested and gave essentially infinitely long transfer times.

We note however that the effective target model predicts significantly more S_2-to-Q_xB850 than S_2-to-Q_xB800 transfer. By comparison of the two transition rates we expect 3.9 times more transfer to B850 than B800, which compares very well with the calculated B850 excess (Figure 7 in the main text) of 3.5.

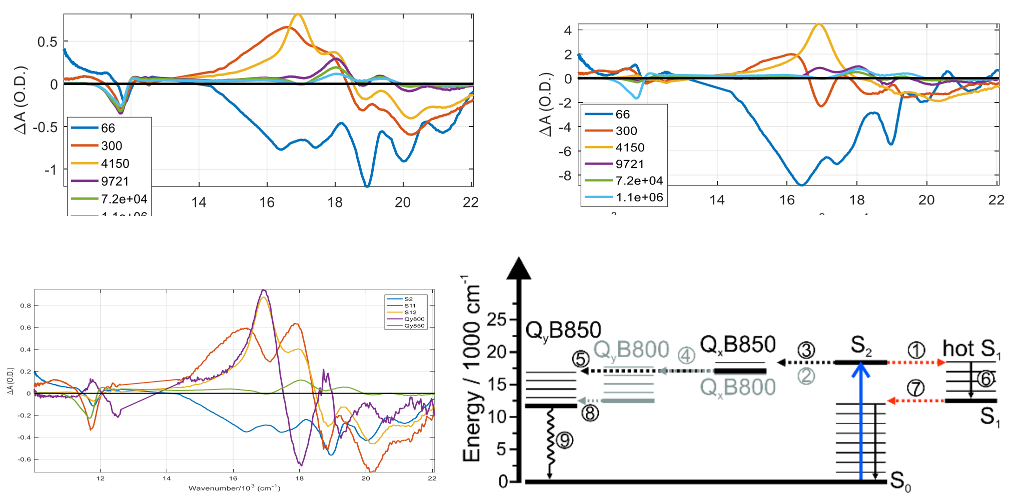

Figure 2: Top left: Target EADS. Top right: Target DAS. Bottom left: raw data. Bottom right: schematic illustration of the target model. Initial excitation is depicted as a blue vertical arrow. Internal conversion processes to and from Q_xB850, Q_xB800, and carotenoid-states are shown as dashed horizontal black, grey, or orange arrows, respectively. Vertical full black arrows denote vibrational relaxation within an electronic state, while the wavy vertical arrows stands for radiative relaxation.

Table 1: Time constants for the relaxation processes in the target model shown in Figure 2

Channel #	1	2	3	4	5	6	7	8	9
time const. (fs)	172	455	116	11	174	338	5877	1308	5.89E+05

ZERO PHONON LINE ESTIMATION FOR THE LYCOPENE S_2 STATE

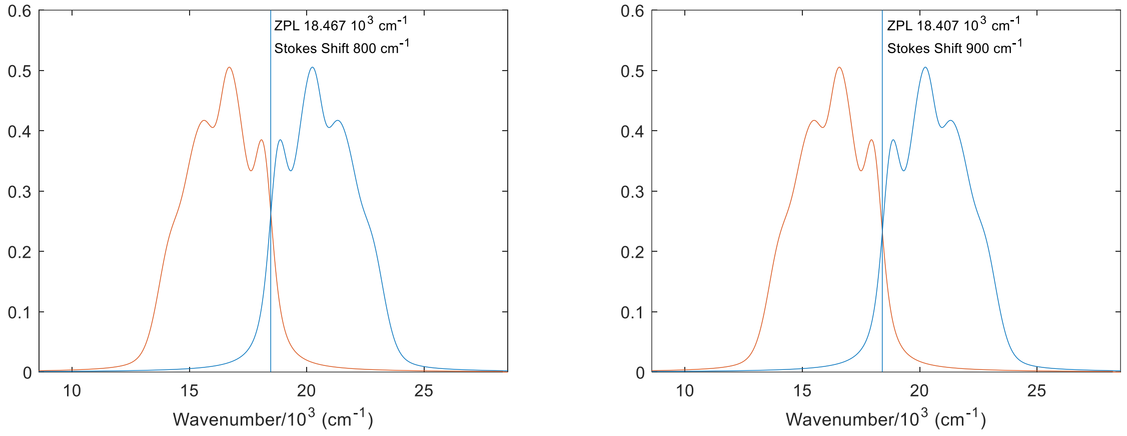

ZPLs are estimated by taking the spectral position of the amplitude maxima of the vibrations in the TA spectra and subtracting their nominal frequency (1150 cm^{-1} and 1530 cm^{-1}, left) or their measured frequency (1100 cm^{-1} and 1460 cm^{-1}; right).

REFERENCES

Damjanovic A, Ritz T, Schulten K (1999) Energy transfer between carotenoids and bacteriochlorophylls in light-harvesting complex II of purple bacteria Phys Rev E 59:3293-3311 doi:DOI 10.1103/PhysRevE.59.3293

Perlik V, Sanda F (2016) Simulations of Coherent Nonlinear Optical Response of Molecular Vibronic Dimers arXiv:160400327 [physicschem-ph]

Perlik V et al. (2015) Vibronic coupling explains the ultrafast carotenoid-to-bacteriochlorophyll energy transfer in natural and artificial light harvesters J Chem Phys 142 doi:10.1063/1.4919548

Rondonuwu FS, Yokoyama K, Fujii R, Koyama Y, Cogdell RJ, Watanabe Y (2004) The role of the 1(1)B(u)(-) state in carotenoid-to-bacteriochlorophyll singlet-energy transfer in the LH2 antenna complexes from Rhodobacter sphaeroides G1C, Rhodobacter sphaeroides 2.4.1, Rhodospirillum molischianum and Rhodopseudomonas acidophila Chem Phys Lett 390:314-322 doi:10.1016/j.cplett.2004.03.089

Tretiak S, Middleton C, Chernyak V, Mukamel S (2000) Bacteriochlorophyll and carotenoid excitonic couplings in the LH2 system of purple bacteria J Phys Chem B 104:9540-9553 doi:10.1021/jp001585m

van Stokkum IHM, Larsen DS, van Grondelle R (2004) Global and target analysis of time-resolved spectra Bba-Bioenergetics 1657:82-104 doi:DOI 10.1016/j.bbabio.2004.04.011
